# Follow-up of children diagnosed with deafness in a neonatal hearing screening program in Manaus

**DOI:** 10.11606/s1518-8787.2022056004207

**Published:** 2022-11-18

**Authors:** João Bosco Lopes Botelho, Diego Monteiro de Carvalho, Giane Zupellari dos Santos-Melo, José Cardoso, Samuel Machado do Nascimento, Wenberger Lanza Daniel de Figueiredo, Larissa Abreu Lacerda, Kristian Holanda Nogueira

**Affiliations:** I Universidade do Estado do Amazonas Faculdade de Medicina Departamento de Medicina Manaus Amazonas Brasil Universidade do Estado do Amazonas. Faculdade de Medicina. Departamento de Medicina. Manaus, Amazonas, Brasil; II Centro Universitário Fametro Faculdade de Medicina Departamento de Medicina Manaus Amazonas Brasil Centro Universitário Fametro. Faculdade de Medicina. Departamento de Medicina. Manaus, Amazonas, Brasil; III Universidade Nilton Lins Faculdade de Medicina Departamento de Medicina Manaus Amazonas Brasil Universidade Nilton Lins. Faculdade de Medicina. Departamento de Medicina. Manaus, Amazonas, Brasil

**Keywords:** Disabled Children, Neonatal Screening, Deafness, diagnosis, Correction of Hearing Impairment, trends, Lost to Follow-Up, Rehabilitation Centers, Health Care Quality, Access, and Evaluation

## Abstract

**OBJECTIVE:**

To evaluate the follow-up of children diagnosed with deafness in neonatal hearing screening and risk factors for hearing loss.

**METHODS:**

Quantitative, cross-sectional, and retrospective study to evaluate factors associated with hearing loss and the follow-up of cases of children diagnosed with audiological dysfunction, by analyzing electronic medical records of 5,305 children referred to a Specialized Center in Type I Rehabilitation, from January/2016 to February/2020, in the city of Manaus, Amazonas. The statistical study used Pearson’s chi-square test and binary logistic regression in which odds ratio scans were obtained with reliability intervals of 95%.

**RESULTS:**

Of the 5,305 children referred for the otoacoustic emission retest, 366 (6.9%) failed the retest. Children diagnosed with sensorineural hearing loss continued in the study, totaling 265 (72.4%). Only 58 (21.9%) children continued in the study to its end, of these 39 had received hearing aids at that point; and 16 (41%) had surgical indication for cochlear implants, of which only 3 (18.7%) had undergone surgery. Among the risk factors for hearing loss, we found 2.6 times more chance of failure in the otoacoustic emissions retest in those children who had a family history of hearing loss and ICU stay.

**CONCLUSION:**

Although the screening flow reaches a large part of live births, the dropout rates during the process are high, therefore, the socioeconomic and geographic characteristics of regions such as the Amazon should be considered as relevant factors to the evasion of rehabilitation programs of these children. Hospitalization in the neonatal ICU and family history of hearing loss in the investigations could be identified as the main and most important factors for alteration of the otoacoustic emissions retests.

## INTRODUCTION

Child deafness has a great impact on the community, whether from an economic or psychosocial point of view. It is the most frequent sensory deficit in humans, with an incidence ranging from 1:300 to 1:1,000 children. According to the World Health Organization (WHO), hearing loss affects about 10% of the world’s population. The frequency of deafness in Brazil is estimated at 4:1,000 births^1^. Hearing impairments may hinder or prevent speech, language, cognition, and socioemotional development from occurring, thus impairing the general cognitive development^2,3^. Early diagnosis is fundamental to minimize these damages.

Neonatal hearing screening (NHS) is an important instrument in implementing hearing conservation programs and detecting hearing disorders early. This procedure is recommended by Brazilian health policies as the first stage of a neonatal hearing health program, which should preferably be performed in the first days of life (24–48 hours), still in maternity and, at most, during the 1st month of life, to minimize the damage caused by this pathology so common in our country.

In the prevention program, at the time of hospital discharge after birth, universal neonatal hearing screening (UNHS) is performed by transient otoacoustic emissions (TOAE) and cochlear-palpebral reflex (CPR) research. If a newborn passes the test, that is, presents a response to TOAE in both ears in the hearing screening test, and has no risk for progressive or retrocochlear loss, an orientation is made on the development of hearing and language and they are discharged. Otherwise, if a newborn fails (when they show no response in one or both ears) the 30-day retest and follow-up by a multidisciplinary team is oriented, aiming at diagnosing this deficiency, to perform periodic evaluations and appropriate interventions for them and their families during the first two years of life^2,4^.

The NHS is part of a set of actions that should be carried out for comprehensive attention to hearing health in childhood: screening, monitoring and follow-up of auditory and language progression, diagnosis, and rehabilitation. Thus, the NHS should be integrated into the network of care for people with disabilities and maternal and child follow-up actions. Articulation, training, and integration with primary care is also extremely important to ensure monitoring and follow-up of the hearing and language development, and for adhesion to referrals to specialized services^4,5^, for example, cochlear implant surgery, which is an effective device for children with severe and/or profound prelingual hearing loss, since it considerably improves the acquisition of oral language via the auditory pathway, which can positively impact other areas of development^9^.

The 2019 Joint Committee on Infant Hearing (JCIH) establishes as a risk indicator for hearing loss (RIHL)^12^: parents’ concern about hearing and speech development; family history of permanent childhood hearing loss; neonatal ICU for more than five days; intrauterine infections such as Cytomegalovirus (CMV), herpes, rubella, syphilis, toxoplasmosis, and more recently Zika; craniofacial malformations; syndromes associated with hearing loss; neurodegenerative disorders; positive cultures in postnatal infections; head trauma; and chemotherapy^12^. The maternal age group equal to or older than 35 years, considered a risk factor for numerous negative outcomes to newborns, has a higher frequency of adverse perinatal outcomes when compared with women aged between 20 and 34 years, with emphasis on prematurity, low birth weight, and low Apgar index, which are risk factors for hearing loss^15^.

In 2012, the Ministry of Health instituted the guidelines for the universal neonatal hearing screening care in the country, in which the rates of performance of the UNHS should be higher than 95% of live births^16^.

However, factors such as lack of family adhesion and the peculiarities of the diagnosis slowed the processes down, preventing most children from reaching the recommended indicators^19^. This makes the medical and speech therapy intervention in the critical period of maturation and functional plasticity of the central nervous system, between six months and two years of age of the child, impossible^20^.

To minimize the damage of hearing loss in the population, the Ministry of Health, via Ordinance no. 2,073, of September 28, 2004, instituted the *Política Nacional de Atenção à Saúde Auditiva* (PNASA – National Policy for Hearing Health Care), promoting wide coverage in the care of patients with hearing impairment in Brazil^23^. Despite the advances in the PNASA implementation process, difficulties persist with the early diagnosis, the agility of hearing aids acquisition, the rehabilitation, and the guarantee of access to user monitoring by the Unified Health System (SUS).

Given the above statement and the reality of the SUS, this study the primarily aimed to describe the follow-up of neonates who showed alterations in the NHS test, and the fraction of neonates who are reevaluated, diagnosed with hearing loss, treated, and followed by a multidisciplinary team.

## METHODS

This is a quantitative, cross-sectional, and retrospective study to evaluate factors associated with hearing loss and the follow-up of cases of children diagnosed with audiological dysfunction, by analyzing electronic medical records from January/2016 to February/2020, in the municipality of Manaus, state of Amazonas. It included children up to two years of age who failed the NHS test in the maternity hospital and were, therefore, referred to a center specialized in Type I rehabilitation (CER I – specialized care of the network of care for people with disabilities within the scope of the SUS), according to Ordinance no. 1,303, from June 28, 2013^24^, to perform the retest, totaling 5,305 medical records analyzed. The 366 cases with retest failure were evaluated according to the ENT diagnosis of hearing loss (Figure). The presence of risk indicators for hearing loss was also considered: gender; family history of hearing loss; gestational complications, such as infections and changes in blood pressure; hospitalization in the Intensive Care Unit; and prematurity, defined as gestational age below 36 weeks.

**Figure:**
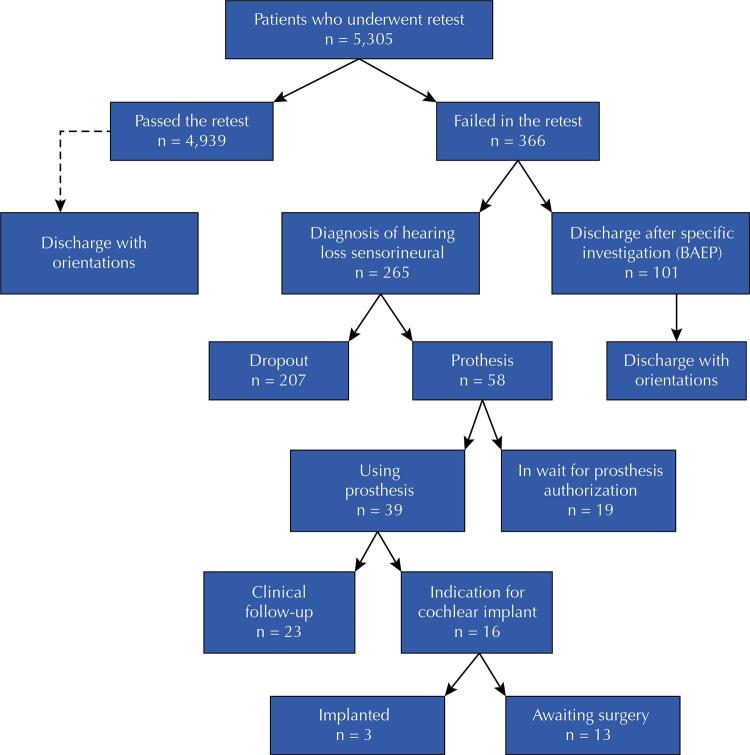
Flowchart of patients referred to the specialized center in rehabilitation I for retest.

Parametric statistical association methods between qualitative variables (chi-square test and binary logistic regression model) were used. The results were interpreted in terms of the statistical significance of the association (p-value) and odds ratios (OR) between the associated factors.

The research followed the recommendations on ethics in studies with human beings and was approved by the Research Ethics Committee of the School of Health Sciences of the Universidade do Estado do Amazonas (CEP/ESA/UEA) under the opinion number 3,827,675 of February 7, 2020.

## RESULTS

In the study period, from January 2016 to February 2020, 148,991 children underwent the otoacoustic emissions test in maternity hospitals in the municipality of Manaus, according to TabNet-Datasus^25^. Of these, 5,305 (3.56%) were referred for diagnostic confirmation of hearing loss in a Specialized Center in Type I Rehabilitation (CER I) in the same city, they comprised 2,989 (56.34%) males and 2,316 (43.66%) females.

Of the 5,305 children submitted to retest in CER I, 4,939 (93.10%) passed and were discharged from the investigative follow-up with proper guidance, whereas 366 (6.9%) failed and proceeded to diagnostic complementation (BAEP – Brainstem Auditory Evoked Potentials), imaging tests (computed tomography scan – CT scan and/or magnetic resonance imaging – MRI of the ear) and therapy (hearing aids and/or CI – cochlear implants). The study followed up the children diagnosed with sensorineural hearing loss, totaling 265 (72.4%). Only 58 (21.9%) children continued in the study to its end; 39 had received hearing aids at that point; and 16 (41%) had surgical indication for cochlear implants, of which only 3 (18.7%) had undergone surgery. A total of 207 (78.1%) children dropped out. At the end, 19 (32.7%) were awaiting release of the hearing aid and 13 (81.3%) were waiting in line for cochlear implant surgery (Figure).

Table 1 shows a significant association between the retest variables with family history, infections/blood pressure, prematurity, and ICU admission (p-value < 0.05), that is, the chance of failure in the retest result was higher for children who presented these risk indicators for hearing loss.

**Table 1:** Risk indicators for hearing loss with the result in the otoacoustic emission retest.

Retest result					

RIHL	Pass	Fail	Total	%	p
Gender					
Male	2,783	206	2,989	56.3	0.981
Female	2,156	160	2,316	43.7	
Total	4,939	366	5,305	100	
Family history					
Present	154	26	180	3.4	0.0001
Absent	4,785	154	5,125	96.6	
Total	4,939	180	5,305	100	
Jaundice					
Present	789	63	852	16.1	0.534
Absent	4,150	303	4,453	83.9	
Total	4,939	366	5,305	100	
Infections/BP					
Present	826	94	920	17.3	0.0001
Absent	4,113	272	4,385	82.7	
Total	4,939	366	5,305	100	
Prematurity					
Present	677	68	745	14	0.01
Absent	4,262	298	4,560	86	
Total	4,939	366	5,305	100	
ICU admission					
Present	364	62	426	8	0.0001
Absent	4,575	304	4,879	92	
Total	4,939	366	5,305	100	

RIHL: risk index for hearing loss; BP: maternal blood pressure; ICU: intensive care unit.

p-values are significant for p < 0.05 – Pearson’s chi-square test.

Among the RIHL, Table 2 shows the chance of failure in the otoacoustic emissions retest was 2.6 times higher in those children who had a family history of hearing loss and ICU stay.

**Table 2:** Odds ratios for categorical predictors.

Odds ratios for categorical predictors			

		Odds ratio	95%CI
Family history Present	Absent	2.618	(1.6986–4.0351)
ICU admission Present	Absent	2.6763	(1.9930–3.5939)

ICU: intensive care unit.

## DISCUSSION

In this study we identified a retest failure of 366 (6.9%) children and a higher prevalence in males, corroborating the studies by Ayas and Yassen (2021)^26^. These authors identified a total of 1,821 newborns. Of those, 81% passed the initial test; 423 (23.22%) were referred for failing the first test and followed up after 2 weeks. Among these babies, 24 (7.03%) failed the retest, of whom, 9 (37.50%) were confirmed with bilateral hearing loss. The incidence of hearing loss in the aforementioned study was 4.94:1,000 and confirmed hearing loss was statistically higher in boys than in girls. Note the importance of exploring a two-stage NHS model to reduce false-positive responses, considering a possible “maturation” of the auditory pathway. The literature already showed suggestions of a delay in the auditory pathway maturation in premature babies compared with full-term newborns. A possible delay in the NHS retest could be considered, in selected cases, significantly saving economic resources and the anxiety of parents, according to the study by Ciorba et al. (2019)^27^ who concluded the BAEP response (and, therefore, of the auditory pathway) went through a possible “maturation” in 1.3% of the infants.

Of the total number of patients referred for the retest, 3,123 (58.9%) showed some risk indicator for hearing loss (RIHL), and some children manifested more than one associated risk factor. Of these evaluated RIHL, 426 children had ICU admission, and 62 (14.5%) failed the retest, with statistical significance (p = 0.0001) (Table 1). According to JCIH (2019)^12^ this risk factor is well known and studied mainly when the ICU stay is longer than five days and involves the need for artificial respiration. And this relationship is established due to diseases, metabolic disorders, and iatrogenesis during the period of intensive care. The specific length of stay in the ICU was not detailed in the cases studied.

Family history of hearing loss is also related to failure of the test. At the Clinical Hospital of Pernambuco, Griz et al. (2010)^28^ demonstrated an OR of 1.20 with 787 neonates and infants, whereas the study by Barboza et al. (2013)^29^ revealed an OR of 1.14. In this study, the family history of hearing loss was present in 180 children; of these 26 (14.4%) failed the otoacoustic emission retest, with a significant association between the variables family history and retest failure, with p = 0.0001 (Table 1).

Hyperbilirubinemia is one of the conditions most associated with hearing loss in ICU patients. Tiensoli et al. (2007)^30^ found that 15% of patients with hearing test alteration had hyperbilirubinemia, whereas this proportion was 0.2% in the group without hearing impairment. Rechia et al. (2016)^31^ also found a significant association between hyperbilirubinemia in children hospitalized in neonatal ICU and failure in the retest. Neonatal jaundice and retest failure showed no statistical significance in this study (p = 0.534) (Table 1).

Prematurity is an important risk factor for congenital hearing loss, especially in neonates with less than 1,500 g at birth. Barboza et al. (2013)^29^, Onoda et al. (2011)^32^, Escobar-Ipuz et al. (2019)^33^, and Marinho et al. (2020)^6^ show that prematurity is the main RIHL in the studied population, on the other hand, the study by Oliveira et al. (2015)^34^ did not observe this association. Our study reported prematurity in 745 (14%) of the children; 68 (9.1%) failed the retest, corroborating, alongside the cited studies, this evidence of association between the variables prematurity and failure in the retest (p = 0.01).

Infections during pregnancy are well-established risk factors in the literature for hearing loss, especially when they are one of the STORCH infections (syphilis, toxoplasmosis, rubella, cytomegalovirus, and herpes). Urinary tract infection is common in young women and represents the most frequent clinical complication during pregnancy. Thus, newborns and infants of mothers who had urinary infections can be considered at risk for hearing loss, not by the infection itself, but in case the treatment used ototoxic medication^28^. In this study, most of the infectious events during pregnancy were urinary tract infections.

Prenatal follow-up, an important preventive care strategy in pregnant women and children, can guide the promotion of health and well-being, besides opportunities to treat problems that can affect mothers and their children during this period. Neonatal infections and changes in maternal blood pressure also showed statistically significant, with p = 0.0001, association with failing the retest (Table 1).

This study also observed that children with indicators of family history of hearing loss and ICU stay were 2.6 times more likely to fail the retest (Table 2). Since neonatal ICU admission is an easily identifiable risk factor, this group of patients should receive special attention regarding their adherence to diagnostic follow-up.

In fact, the noticeably high prevalence throughout the series of dropouts during the follow-up of these children after confirmation of the diagnosis of sensorineural hearing loss stands out. Although this study did not measure the number of absences in the retest, considering the initial number of referrals is unknow, we observed problems in the clinical conduction in which 207 (78.1%) children who failed the retest and performed diagnostic confirmation dropped out during the follow-up.

According to Samelli et al. (2019)^35^, regardless of the management model (Family Health Strategy and traditional basic health unit), low scores were found in response to the Primary Care Assessment Tool (PCATool) child version questionnaire – which assesses the quality of health services, measuring aspects of structure, process, and result, relating the users’ experiences with the professionals and the health service, measuring their satisfaction. Parents evaluated some points as unsatisfactory, such as accessibility, comprehensiveness, and family orientation. This performance can negatively affect the quality and integrality of care to the at-risk baby. In this research, the factors reported in the medical records about the discontinuity of follow-up were social issues, such as a chemically dependent mother, a change of home residence, a dissatisfaction with the quality of primary health care services, the geographic characteristics of our region, and the financial difficulties to abide by the follow-up.

Among the factors that lead to dropout are: low schooling level of mothers, who are unaware of the importance of hearing screening and low socioeconomic status^36,37^, since some mothers claim not to have enough money for bus tickets. The author identified 20% of absence in the retest in the neonatal unit of the University Hospital of the municipality of Maringá, state of Paraná, a similar situation to the one in the work of Berni et al. (2010)^38^ when studying the NHS in a public hospital, located in the municipality of Campinas, state of São Paulo, absences to retest was 24.8%.

About half of the children with alterations in the initial otoacoustic emissions test lacked an adequate follow-up to confirm their diagnosis^12^.

Among those who maintained the follow-up and confirmed the sensorineural deafness diagnosis, with indication of the hearing aid device, 19 (32.7%) were not yet in use, since they were waiting for the prosthesis to be authorized. Of the 39 children using hearing aids, 16 (41%) had surgical indication of cochlear implant (CI) but only 3 (18.75%) could undergo the surgery until the end of data collection (performed outside the state of Amazonas), the other 13 (81.25%) were still in the waiting list of the regulation system (SISREG) waiting for authorization to perform preoperative imaging and diagnostic examinations (Figure).

Part of the justification for the slow progression to surgery seems to be related to the extensive imaging investigation protocol (tomographies and resonances under sedation) and the administrative process for approving tickets, lodging, and scheduling consultations/surgical evaluation to be performed, mainly in the State of São Paulo, via the public service in the SUS program *Tratamento Fora de Domicílio* (TFD – Out-of-Home District Treatment) of the state of Amazonas Department of Health.

Regarding the follow-up for CI surgery, we cite as an example some cases that may explain the social reasons for non-progression in therapy: the first, one of the children who came from the municipality of São Gabriel da Cachoeira, state of Amazonas (852 km away from Manaus), and belonged to an indigenous ethnic group. Born premature, with late diagnosis of hearing loss at 2 years of age already in irregular use of hearing aids, with indication for CI surgery, the case reported the difficulties of access to speech therapy even with the hearing aid. The second child was from the rural area of Itacoatiara, state of Amazonas (270 km away from Manaus), whose family lived on subsistence agriculture. The child also presented social and financial difficulties to attend consultations and speech therapy, which is why she did not go to surgery. Two other children came from the outskirts of the city of Manaus and, despite living in the capital, both had a social context of risk, ranging from involvement of parents with illicit activities to reports of theft of the hearing aids.

Within this context, the Amazon sociogeographic situation is an important issue for consideration when planning the promotion and implementation of public policies of auditory health, especially in the child population. In cases where the enormous distances are the obstacle, we have other barriers such as situations of crime and social vulnerability that hinder access to rehabilitation treatment.

Lanzetta (2008)^39^, when studying the follow-up of children with hearing loss regarding the implantation of hearing aids, noticed that only 26% sought the service spontaneously. When social services requested the follow-up, this number reached 44%. These findings reinforce the importance of the parents’ adhesion to the hearing screening program and subsequent follow-up, besides revealing the need for a neonatal hearing screening program including a work front with social workers who can accompany these mothers to reduce the program’s dropout rate.

Note that the Northern region of Brazil has a single cochlear implant center of the SUS, at the Bettina Ferro de Souza University Hospital, in the municipality of Belém, state of Pará, and that, despite being in the same region, due to the continental Brazilian dimensions, to the internal demand of the state of Pará, and to the TFD policy adopted in the state of Amazonas, displacement remains a major obstacle to these cases.

Although we observed a broad coverage of neonatal hearing screening, the follow-up difficulties, especially for CI surgery, should be reevaluated with public policies designed specifically for the region, considering social, economic, and geographic challenges.

In conclusion, although the screening flow reaches a large part of live births, the dropout rates during the process are high, therefore, the socioeconomic and geographic characteristics of regions such as the Amazon should be considered as relevant factors to these children dropping out of rehabilitation programs. Hospitalization in the neonatal ICU and family history of hearing loss in the investigations could be identified as the main and most important factors for alteration of the otoacoustic emissions retests.
