# Supplementary material for: Follow-up of children diagnosed with deafness in a neonatal hearing screening program in Manaus
Source: Rev Saude Publica. 2022 Nov 18;56:120. doi: 10.11606/s1518-8787.2022056004207 (PMC9749742; doi:10.11606/s1518-8787.2022056004207)
Supplement: Translation

# Translation: Seguimento de crianças com diagnóstico de surdez em programa de triagem auditiva neonatal em Manaus

**Authors:** João Bosco Lopes Botelho, Diego Monteiro de Carvalho, Giane Zupellari dos Santos-Melo, José Cardoso, Samuel Machado do Nascimento, Wenberger Lanza Daniel de Figueiredo, Larissa Abreu Lacerda, Kristian Holanda Nogueira

**Affiliations:** I Brasil Universidade do Estado do Amazonas. Faculdade de Medicina. Departamento de Medicina. Manaus, Amazonas, Brasil; II Brasil Centro Universitário Fametro. Faculdade de Medicina. Departamento de Medicina. Manaus, Amazonas, Brasil; III Brasil Universidade Nilton Lins. Faculdade de Medicina. Departamento de Medicina. Manaus, Amazonas, Brasil

**Keywords:** Crianças com Deficiência, Triagem Neonatal, Surdez, diagnóstico, Correção de Deficiência Auditiva, tendências, Perda de Seguimento, Centros de Reabilitação, Qualidade, Acesso e Avaliação da Assistência à Saúde

## Abstract

**OBJETIVO:**

Avaliar o seguimento das crianças com diagnóstico de surdez na triagem auditiva neonatal e fatores de risco para deficiência auditiva.

**MÉTODOS:**

Estudo quantitativo, transversal e retrospectivo para avaliação de fatores associados à perda auditiva e o seguimento dos casos de crianças diagnosticadas com disfunção audiológica, por meio da análise de prontuários eletrônicos de 5.305 crianças encaminhadas a um Centro Especializado em Reabilitação Tipo I, no período de janeiro/2016 a fevereiro/2020, na cidade de Manaus, Amazonas. O estudo estatístico utilizou o teste qui-quadrado de Pearson e por regressão logística binária nos quais foram obtidos *odds ratio* com intervalos de confiabilidade de 95%.

**RESULTADOS:**

Das 5.305 crianças encaminhadas para realização do reteste da orelhinha, 366 (6,9%) falharam no reteste. Prosseguiram no estudo as crianças com diagnóstico de perda auditiva neurossensorial, totalizando 265 (72,4%). Permanecendo, no final da pesquisa, apenas 58 (21,9%) crianças, destas 39 receberam aparelho auditivo até o presente estudo; e 16 (41%) já tinham indicação cirúrgica para implante coclear, sendo que apenas três (18,7%) haviam realizado a cirurgia. Dentre os fatores de risco para deficiência auditiva encontramos 2,6 vezes mais chance de falha no reteste da orelhinha naquelas crianças que tinham história familiar de perda auditiva e internação em UTI.

**CONCLUSÕES:**

Embora o fluxo de triagem alcance boa parte dos nascidos vivos, as taxas de evasão durante o processo são altas, portanto, as características socioeconômicas e geográficas de regiões como a Amazônia devem ser consideradas como fatores relevantes à evasão dos programas de reabilitação dessas crianças. Foi possível identificar que a internação em UTI neonatal e o histórico familiar de perda auditiva presentes nas investigações compõem os principais e mais importantes fatores para alteração dos retestes da orelhinha.

## INTRODUÇÃO

A surdez infantil exerce um grande impacto sobre a comunidade, seja do ponto de vista econômico ou psicossocial. É o déficit sensorial mais frequente em humanos, com incidência que varia de 1:300 a 1:1.000 crianças. Segundo a Organização Mundial da Saúde (OMS), a perda auditiva atinge cerca de 10% da população mundial. A frequência da surdez no Brasil é estimada em 4:1.000 nascimentos^1^. Deficiências auditivas podem dificultar ou impedir que o desenvolvimento da fala, linguagem, cognição e desenvolvimento socioemocional ocorra, prejudicando, assim, o desenvolvimento cognitivo geral^2,3^. A importância de um diagnóstico precoce é fundamental para minimizar esses danos.

A triagem auditiva neonatal (TAN) é um importante instrumento na implantação de programas de conservação da audição e na detecção precoce das alterações auditivas. Esse procedimento é recomendado pelas políticas de saúde brasileiras como a primeira etapa de um programa de saúde auditiva neonatal, que, preferencialmente, deve ser realizado nos primeiros dias de vida (24–48 horas), ainda na maternidade e, no máximo, durante o 1º mês de vida, para que possamos minimizar os prejuízos causados por esta patologia tão comum em nosso meio.

No programa de prevenção, por ocasião da alta hospitalar após o nascimento, é realizada a triagem auditiva neonatal universal (TANU) por meio de emissões otoacústicas transientes (EOAT) e pesquisa do reflexo cócleo palpebral (RCP). Se o neonato passa, ou seja, apresenta resposta às EOAT em ambas as orelhas no teste da triagem auditiva, e não tem riscos para perda progressiva ou retrococlear, é feita uma orientação sobre o desenvolvimento da audição e da linguagem e ele recebe alta. Caso contrário, se há falha (quando não apresenta resposta em uma ou ambas as orelhas) é orientado o reteste com 30 dias e acompanhamento por uma equipe multidisciplinar, com o objetivo de encaminhá-los para diagnóstico desta deficiência, para realizar avaliações periódicas e intervenções adequadas a eles e seus familiares, durante os dois primeiros anos de vida^2,4^.

A TAN faz parte de um conjunto de ações que devem ser realizadas para a atenção integral à saúde auditiva na infância: triagem, monitoramento e acompanhamento da progressão auditiva e da linguagem, diagnóstico e reabilitação. Desta forma, a TAN deve estar integrada à rede de cuidados à pessoa com deficiência e às ações de acompanhamento materno-infantil. Também é de extrema importância a articulação, capacitação e integração com a atenção básica para garantir o monitoramento e acompanhar o desenvolvimento da audição e da linguagem, e para a adesão aos encaminhamentos para serviços especializados^4,5^, por exemplo, a cirurgia de implante coclear, o qual é um dispositivo eficaz para crianças com perda auditiva pré-lingual severa e/ou profunda, pois proporciona considerável melhora na aquisição da linguagem oral por meio da via auditiva, o que pode ter um impacto positivo em outras áreas do desenvolvimento^9^.

O *Joint Committee on Infant Hearing* (JCIH) de 2019 estabelece como indicador de risco para deficiência auditiva (IRDA)^12^: preocupação dos pais com relação ao desenvolvimento da audição e fala; histórico familiar de perda auditiva infantil permanente; UTI neonatal por mais de cinco dias; infecções intrauterinas, como Citomegalovírus (CMV), herpes, rubéola, sífilis, toxoplasmose e mais recentemente o Zika; malformações craniofaciais; síndromes associadas à perda auditiva; desordens neurodegenerativas; culturas positivas em infecções pós-natal; trauma cranioencefálico e quimioterapia^12^. A faixa etária materna igual ou superior a 35 anos, considerada fator de risco para inúmeros desfechos negativos relacionados aos recém-nascidos, têm maior frequência de resultados perinatais adversos quando comparada às mulheres com idade entre 20 e 34 anos, com destaque para prematuridade, baixo peso ao nascer e baixo índice de Apgar, sendo fatores de risco para perda auditiva^15^.

Em 2012, o Ministério da Saúde instaurou as diretrizes de atenção da triagem auditiva neonatal universal no país, em que os índices de realização da TANU devem ser superiores a 95% dos nascidos vivos^16^.

Contudo, fatores como a não adesão da família e as peculiaridades do diagnóstico retardaram os processos, impedindo que os indicadores preconizados fossem alcançados, na maior parte das crianças^19^. O que impossibilita a intervenção médica e fonoaudiológica no período crítico de maturação e plasticidade funcional do sistema nervoso central, entre os seis meses e dois anos de idade da criança^20^.

Com vistas a minimizar o dano da deficiência auditiva na população, o Ministério da Saúde, por meio da Portaria n. 2.073, de 28 de setembro de 2004, instituiu a Política Nacional de Atenção à Saúde Auditiva (PNASA), promovendo a ampla cobertura no atendimento aos pacientes portadores de deficiência auditiva no Brasil^23^. Apesar dos avanços no processo de implementação da PNASA, persistem dificuldades com diagnóstico precoce, a agilidade da aquisição das próteses auditivas, a reabilitação e da garantia do acesso ao acompanhamento do usuário pelo Sistema Único de Saúde.

Mediante a afirmativa acima e da realidade do SUS, o objetivo primário desse estudo foi descrever o seguimento dos neonatos que apresentaram alteração no teste de TAN, bem como a fração de neonatos que são reavaliados, diagnosticados com perda auditiva, tratados e acompanhados por uma equipe multidisciplinar.

## MÉTODOS

Trata-se de um estudo quantitativo, transversal e retrospectivo para avaliação de fatores associados à perda auditiva e o seguimento dos casos de crianças diagnosticadas com disfunção audiológica, por meio da análise de prontuários eletrônicos no período de janeiro/2016 a fevereiro/2020, na cidade de Manaus, Amazonas. Foram incluídas crianças de até dois anos de idade com falha no teste de TAN na maternidade e, portanto, encaminhados a um centro especializado em reabilitação Tipo I (CER I - atenção especializada da rede de cuidados à pessoa com deficiência no âmbito do SUS), segundo a Portaria n. 1.303, de 28 de junho de 2013^24^, para realização do reteste, totalizando 5.305 prontuários analisados. Os 366 casos com falha no reteste foram avaliados segundo o diagnóstico otorrinolaringológico de perda auditiva (Figura).Considerou-se, ainda, a presença dos indicadores de risco para deficiência auditiva: sexo, histórico familiar de perda auditiva, intercorrências gestacionais, como infecções e alterações na pressão arterial, internação em Unidade de Terapia Intensiva e prematuridade, definida como idade gestacional inferior a 36 semanas.

**Figura:** Fluxograma dos pacientes encaminhados ao centro especializado em reabilitação I para reteste.

Foram utilizados métodos estatísticos paramétricos de associação entre as variáveis qualitativas (teste qui-quadrado e modelo de regressão logística binária). Os resultados foram interpretados em termos da significância estatística da associação (p-valor) e das razões de chances *(odds ratio)* entre os fatores associados.

A pesquisa seguiu as recomendações sobre ética em estudos com seres humanos, sendo aprovado pelo Comitê de Ética em Pesquisa da Escola Superior de Ciências da Saúde da Universidade do Estado do Amazonas (CEP/ESA/UEA) sob o número de parecer 3.827.675 de 07 de fevereiro de 2020.

## RESULTADOS

No período do estudo, de janeiro de 2016 a fevereiro de 2020, 148.991 crianças realizaram o teste da orelhinha nas maternidades da cidade de Manaus, segundo TabNet-Datasus^25^. Dessas, 5.305 (3,56%) foram encaminhadas para confirmação diagnóstica de perda auditiva em um Centro Especializado em Reabilitação Tipo I (CER I) na mesma cidade, sendo 2.989 (56,34%) do sexo masculino e 2.316 (43,66%) do sexo feminino.

Das 5.305 crianças submetidas ao reteste no CER I, 4.939 (93,10%) passaram e receberam alta do seguimento investigativo com as devidas orientações, enquanto 366 (6,9%) não passaram e seguiram para complementação diagnóstica (PEATE – Potencial Evocado Auditivo de Tronco Encefálico), exames de imagens (tomografia computadorizada – TC e/ou ressonância magnética – RM de ouvido) e terapia (AASI – aparelho de amplificação sonora individual e/ou IC – implante coclear). Para o interesse do estudo permaneceram as crianças com diagnóstico de perda auditiva neurossensorial que totalizaram 265 (72,4%). Destas, prosseguiram no estudo apenas 58 (21,9%) crianças; 39 tinham recebido aparelho auditivo até o presente estudo; e 16 (41%) já tinham indicação cirúrgica para implante coclear, sendo que apenas três (18,7%) haviam realizado a cirurgia. Ocorreu evasão em 207 (78,1%) das crianças. 19 (32,7%) aguardavam liberação do aparelho auditivo e 13 (81,3%) seguiam na fila de espera para realização da cirurgia de implante coclear (Figura).

Na Tabela 1 encontramos associação significativa entre as variáveis reteste com história familiar, infecções/PA, prematuridade e internação em UTI (p-valor < 0.05), ou seja, a chance de falha no resultado do reteste foi maior para as crianças que apresentaram esses indicadores de risco para deficiência auditiva.

**Tabela 1:** Indicadores de risco para deficiência auditiva com o resultado no reteste da orelhinha.

Resultado do reteste					

IRDA	Passou	Falhou	Total	%	p
Sexo					
Masculino	2.783	206	2.989	56,3	0,981
Feminino	2.156	160	2.316	43,7	
Total	4.939	366	5.305	100	
História familiar					
Presente	154	26	180	3,4	0,0001
Ausente	4.785	154	5.125	96,6	
Total	4.939	180	5.305	100	
Icterícia					
Presente	789	63	852	16,1	0,534
Ausente	4.150	303	4.453	83,9	
Total	4.939	366	5.305	100	
Infecções/PA					
Presente	826	94	920	17,3	0,0001
Ausente	4.113	272	4.385	82,7	
Total	4.939	366	5.305	100	
Prematuridade					
Presente	677	68	745	14	0,01
Ausente	4.262	298	4.560	86	
Total	4.939	366	5.305	100	
Internação UTI					
Presente	364	62	426	8	0,0001
Ausente	4.575	304	4.879	92	
Total	4.939	366	5.305	100	

IRDA: índice de risco para deficiência auditiva; PA: pressão arterial materna; UTI: unidade de terapia intensiva.

Valores de p são significativos para p < 0,05 – teste qui-quadrado de Pearson.

Dentre os indicadores de risco para deficiência auditiva (IRDA) encontramos 2,6 vezes mais chance de falha no reteste da orelhinha naquelas crianças que tinham história familiar de perda auditiva (2,618) e internação em UTI (2,6763), conforme Tabela 2.

**Tabela 2:** Razões de chances para preditores categóricos.

Razões de chances para preditores categóricos			

		Razão de chance	IC95%
**História familiar** presente	Ausente	2,618	(1,6986–4,0351)
**Internação UTI** presente	Ausente	2,6763	(1,9930–3,5939)

UTI: unidade de terapia intensiva.

## DISCUSSÃO

Neste estudo identificamos uma falha no reteste de 366 (6,9%) crianças e uma prevalência maior no sexo masculino, corroborando os estudos de Ayas e Yassen^26^ (2021). Esses autores identificaram um total de 1.821 recém-nascidos. Foram aprovados no teste inicial 81% dos bebês; 423 (23,22%) foram encaminhados por falharem ao primeiro teste e acompanhados após 2 semanas. Entre esses bebês, 24 (7,03%) falharam no reteste. Nove (37,50%) dos 24 bebês foram confirmados com perda auditiva bilateral. A incidência de perda auditiva no estudo supracitado foi de 4,94:1.000 e a perda auditiva confirmada foi estatisticamente maior em meninos do que em meninas. Vale ressaltar a importância de explorar um modelo de TAN de dois estágios para reduzir as respostas falso-positivas, levando em consideração uma possível “maturação” da via auditiva. Já foi sugerido na literatura um atraso na maturação da via auditiva em bebês prematuros em comparação com recém-nascidos a termo. Um possível atraso no reteste da TAN poderia ser considerado, em casos selecionados, com economia significativa de recursos econômicos e ansiedade dos pais, conforme o estudo de Ciorba et al. (2019)^27^ que concluíram uma possível “maturação” da resposta do PEATE (e, portanto, da via auditiva) em 1,3% dos lactentes.

Do total de pacientes encaminhados para realização do reteste da orelhinha, 3.123 (58,9%) apresentavam algum indicador de risco para deficiência auditiva (IRDA), e algumas crianças manifestavam mais de um fator de risco associado. Destes IRDA avaliados, a internação em UTI foi observada em 426 crianças, sendo que 62 (14,5%) falharam no reteste, com significância estatística (p = 0,0001) (Tabela 1). Segundo JCIH^12^(2019) este fator de risco é bem conhecido e estudado principalmente quando a permanência é superior a cinco dias na UTI e envolve a necessidade de respiração artificial. E essa relação se estabelece devido a doenças, distúrbios metabólicos e iatrogenias durante o período de cuidados intensivos. Não houve detalhamento do tempo específico de permanência na UTI dos casos estudados.

História familiar de perda auditiva também se relaciona com falha no teste da orelhinha. No Hospital das Clínicas de Pernambuco, Griz et al. (2010)^28^ com 787 neonatos e lactentes, demonstrou uma OR de 1,20 enquanto o estudo de Barboza et al. (2013)^29^ revelou uma OR de 1,14. Neste trabalho, a história familiar de perda auditiva esteve presente em 180 crianças; destas 26 (14,4%) tiveram falha no reteste da orelhinha havendo associação significativa entre as variáveis histórico familiar e falha no reteste, com p = 0,0001 (Tabela 1).

A hiperbilirrubinemia é uma das condições mais associadas com perda auditiva em crianças internadas em UTI. Tiensoli et al. (2007)^30^ encontrou que 15% dos pacientes com alteração no teste auditivo tinham hiperbilirrubinemia, enquanto no grupo sem alteração auditiva essa proporção foi de 0,2%. Rechia et al. (2016)^31^ também encontrou associação significativa entre hiperbilirrubinemia em crianças internadas em UTI neonatal e falha no reteste da orelhinha. Não houve significância estatística entre icterícia neonatal e falha no reteste, nesta pesquisa (p = 0,534) (Tabela 1).

Sobre a prematuridade trata-se de importante fator de risco para perda auditiva congênita, notadamente em neonatos com menos de 1.500g ao nascer. Barboza et al. (2013)^29^, assim como em Onoda et al. (2011)^32^, Escobar-Ipuz et al. (2019)^33^ e Marinho et al. (2020)^6^ demonstram ser a prematuridade a principal IRDA na população estudada, em contrapartida essa associação não foi observada no estudo de Oliveira et al. (2015)^34^. Neste estudo a prematuridade foi relatada em 745 (14%) das crianças; 68 (9,1%) apresentaram falha no reteste, corroborando com os estudos citados, essa evidência de associação entre as variáveis prematuridade e falha no reteste (p = 0,01).

Infecções durante a gestação são fatores de risco bem estabelecidos na literatura para perda auditiva, principalmente quando são uma das STORCH (sífilis, toxoplasmose, rubéola, citomegalovírus e herpes). A infecção do trato urinário é comum em mulheres jovens e representa a complicação clínica mais frequente no período gestacional. Isso faz com que os neonatos e lactentes das mães que tiveram infecções urinárias possam ser considerados de risco para perda auditiva, não pela própria infecção, mas no caso de ter havido tratamento por medicação ototóxica^28^. Neste estudo, grande parte dos eventos infecciosos durante a gestação foram infecções do trato urinário.

O acompanhamento pré-natal, estratégia importante de cuidados preventivos em gestantes e crianças, é capaz de orientar a promoção da saúde e do bem-estar, além de oportunizar o tratamento de problemas que podem afetar as mães e seus filhos nesse período. Infecções neonatais e alteração na pressão arterial materna também teve significância estatística, com p = 0,0001, associados à falha no reteste da orelhinha (Tabela 1).

Neste trabalho foi observado, ainda, que as crianças com indicadores de história familiar de perda auditiva e internação em UTI apresentavam 2,6 vezes mais chances de falha no reteste da orelhinha (Tabela 2). Como a internação em UTI neonatal é um fator de risco facilmente identificável, importa haver uma atenção especial a esse grupo de pacientes em relação a sua aderência ao seguimento diagnóstico.

De fato, chama a atenção, em toda a casuística, a alta prevalência de evasão durante o seguimento dessas crianças após confirmação do diagnóstico de perda auditiva neurossensorial. Embora este trabalho não tenha aferido o número de faltas ao reteste, tendo em vista desconhecermos o quantitativo inicial de encaminhamentos, notou-se problemas na adesão à condução clínica em que 207 (78,1%) crianças que falharam no reteste e realizaram a confirmação diagnóstica evadiram durante o seguimento.

Segundo Samelli et al. (2019)^35^, independente do modelo de gestão (Estratégia Saúde da Família e unidade básica de saúde tradicional), verificou-se baixos escores, em resposta ao questionário PCATool versão criança – o qual avalia a qualidade dos serviços de saúde, mensurando aspectos de estrutura, processo e resultado, relacionando as experiências do usuário com os profissionais e com o serviço de saúde, medindo sua satisfação. Os pais avaliaram como insatisfatórios alguns pontos, como acessibilidade, integralidade e orientação familiar. Esse desempenho pode trazer consequências negativas para a qualidade e integralidade do cuidado ao bebê de risco. Nesta pesquisa, os fatores relatados nos prontuários sobre a descontinuidade do seguimento foram questões sociais, como mãe dependente química, mudança de residência domiciliar, insatisfação com a qualidade dos serviços de atenção primária à saúde, características geográficas da nossa região e dificuldades financeiras em seguir o acompanhamento.

Entre os fatores que levam à evasão estão: baixo grau escolar das mães, que não se conscientizam da importância da triagem auditiva e o baixo nível socioeconômico^36,37^, visto que algumas mães alegam não ter dinheiro suficiente para passagens de ônibus. O autor identificou 20% de falta ao reteste na unidade neonatal do Hospital Universitário da cidade de Maringá, PR, situação semelhante encontrada no trabalho de Berni et al (2010)^38^ ao estudar a TAN em um hospital da rede pública, situado na cidade de Campinas, SP, encontrou 24,8% de faltas ao reteste.

Cerca de metade das crianças que apresentam alteração no teste inicial da orelhinha não têm um adequado seguimento para confirmação de seu diagnóstico^12^.

Já, entre aquelas que mantiveram o seguimento e confirmaram o diagnóstico de surdez neurossensorial, com indicação do uso do aparelho de amplificação sonora individual (AASI), 19 (32,7%) ainda não estavam em uso, pois aguardavam liberação da prótese. Das 39 crianças usuárias de AASI, 16 (41%) tiveram indicação cirúrgica de implante coclear (IC), porém, apenas 3 (18,75%) conseguiram realizar a cirurgia até o final da coleta de dados (realizada fora do estado do Amazonas), as outras 13 (81,25%) seguiam na fila de espera do sistema de regulação (SISREG) aguardando autorização para realizar exames de imagem pré-operatórios e diagnóstico (Figura).

Parte da justificativa para a morosidade na progressão para a cirurgia parece estar relacionada ao extenso protocolo de investigação imagenológica (tomografias e ressonâncias sob sedação) e o processo administrativo para liberação de passagens, hospedagem e agendamento de consultas/avaliação cirúrgica a serem realizadas, principalmente no Estado de São Paulo, via serviço público no programa SUS da Secretaria Estadual de Saúde do Amazonas de Tratamento Fora de Domicílio (TFD).

Sobre o seguimento para a cirurgia do IC, citamos como exemplo alguns casos que podem explicar as razões sociais da não progressão na terapia: a primeira, uma das crianças que era proveniente do município de São Gabriel da Cachoeira, AM (852 km até Manaus), e pertencia a uma etnia indígena. Nascida de parto prematuro, com diagnóstico tardio de perda auditiva aos 2 anos de idade já em uso irregular do AASI, com indicação para cirurgia de IC, relatava as dificuldades de acesso ao acompanhamento fonoterápico mesmo com o AASI. A segunda criança era da zona rural de Itacoatiara, AM (270 km até Manaus), cuja família vivia de agricultura de subsistência. Ela também apresentava dificuldades sociais e financeiras para comparecimento às consultas e à fonoterapia, razão pela qual não seguiu para a cirurgia. Ainda, outras duas eram oriundas da periferia da cidade de Manaus e, apesar de habitarem na capital, o contexto social de ambas era de risco, havendo desde envolvimento dos pais com atividades ilícitas até relato de furto do AASI.

Dentro desse contexto, a situação sociogeográfica amazônica é uma questão importante a ser considerada ao se planejar a promoção e execução de políticas públicas de saúde auditiva, em especial na população infantil. Nos casos em que as enormes distâncias são o obstáculo, temos outras barreiras como situações de criminalidade e vulnerabilidade social que dificultam o acesso ao tratamento de reabilitação.

Lanzetta^39^ (2008) ao estudar o acompanhamento de crianças com perda auditiva quanto à implantação de AASI, percebeu que apenas 26% procuraram o serviço espontaneamente. Quando o serviço social solicitava o retorno, este número chegava a 44%. Estes achados reforçam a importância da adesão dos pais ao programa de triagem auditiva e posterior acompanhamento, além de revelar a necessidade de um programa de triagem auditiva neonatal incluindo uma frente de trabalho com assistentes sociais que possam acompanhar essas mães com o objetivo de diminuir o índice de evasão do programa.

É válido destacar que a região Norte do Brasil possui um único centro de implante coclear do SUS, localizado no Hospital Universitário Bettina Ferro de Souza, em Belém do Pará, e que embora seja na mesma região, pelas dimensões continentais brasileiras, pela própria demanda interna do estado do Pará e pela política de TFD adotada no estado do Amazonas, o deslocamento continua sendo grande empecilho para estes casos.

Apesar de observarmos uma cobertura ampla para a realização da triagem auditiva neonatal, as dificuldades do seguimento, em especial para a cirurgia do IC, deverão ser repensadas por meio de políticas públicas desenhadas especificamente para a região, considerando os desafios sociais, econômicos e geográficos.

Conclui-se que, embora o fluxo de triagem alcance boa parte dos nascidos vivos, as taxas de evasão durante o processo são altas, portanto, as características socioeconômicas e geográficas de regiões como a Amazônica, devem ser consideradas como fatores relevantes à evasão dos programas de reabilitação dessas crianças. Foi possível identificar que a internação em UTI neonatal e o histórico familiar de perda auditiva presentes nas investigações, compõem os principais e mais importantes fatores para alteração de retestes da orelhinha.
